# Using near-infrared spectroscopy to predict nitrogen and phosphorus concentrations of herbarium specimens under different storage conditions

**DOI:** 10.1186/s13007-024-01146-x

**Published:** 2024-02-01

**Authors:** Paul Kühn, Tobias Proß, Christine Römermann, Karsten Wesche, Helge Bruelheide

**Affiliations:** 1 Institute of Ecology and Evolution, Friedrich Schiller University Jena, Philosophenweg 16, 07743 Jena, Germany; 2 German Centre for Integrative Biodiversity Research (iDiv) Halle-Jena-Leipzig, Puschstraße 4, 04103 Leipzig, Germany; 3 Institute of Biology/Geobotany and Botanical Garden, Martin Luther University Halle-Wittenberg, Am Kirchtor 1, 06108 Halle (Saale), Germany; 4 Senckenberg Institute of Plant Form and Function Jena, 07743 Jena, Germany; 5 Botany Department, Senckenberg Museum of Natural History Görlitz, Am Museum 1, 02826 Görlitz, Germany; 6 International Institute Zittau, Technische Universität Dresden, Am Grünen Graben 23, 02826 Görlitz, Germany

**Keywords:** Fertilization, Functional traits, Herbarium, Near-infrared spectroscopy, Nitrogen, Phosphorus

## Abstract

**Background:**

Herbaria are becoming increasingly important as archives of biodiversity, and play a central role in taxonomic and biogeographic studies. There is also an ongoing interest in functional traits and the way they mediate interactions between a plant species and its environment. Herbarium specimens allow tracking trait values over time, and thus, capturing consequences of anthropogenic activities such as eutrophication. Here, we present an open, reproducible, non-destructive workflow to collect leaf trait data from herbarium specimens using near-infrared spectroscopy (NIRS), and a proof of concept for the reliability of this approach.

**Results:**

We carried out three experiments to test the suitability of non-destructive NIRS methods to predict leaf traits both for fresh and dried leaves: (1) With a fertilization experiment, we studied whether NIRS was able to capture changes in leaf N and leaf P during a fertilization experiment and we compared contents predicted by NIRS with results obtained from regular wet lab methods. Calibration models for leaf nitrogen and phosphorus contents had a quality of R^2^ = 0.7 and 0.5, respectively. We fitted calibration models for NIRS readings on fresh and dried leaf samples, both of which produced equally precise predictions compared to results from wet lab analyses. (2) We tested the effect of herbarium conservation on NIRS readings by simulating them through the application of six treatments combining freezing, drying and pesticide spraying in a factorial scheme and comparing these with untreated samples. No consistent changes were observed in the spectra quality before and after the simulated herbarium conditions. (3) Finally, we studied the effect of specimen storage duration using specimens from a 2018 study which were re-analyzed and compared with spectra obtained in 2021. No consistent changes in spectra were observed after the storage period.

**Conclusions:**

The results demonstrate the reliability of NIRS to measure leaf N and P on herbarium samples. Together with the calibration method and dataset presented here, they provide a toolset allowing researchers to study the development of leaf traits and their response to environmental changes over decades and even centuries in a fast and non-destructive manner.

**Supplementary Information:**

The online version contains supplementary material available at 10.1186/s13007-024-01146-x.

## Background

Natural history collections are increasingly used in ecological research. Herbaria in particular have been centers of taxonomic and systematic studies since long, and are also appreciated for their usefulness in revealing species distributions [1]. In a historical perspective, studies of biogeography use herbarium specimens as evidence for changes in range size [2], and phenology studies compare phenological stages of species between different times and locations [3]. Studies of functional traits retrieved from herbarium specimens give insights into the trade-offs and changes related to the plant leaf economics spectrum and plant fitness, which in turn can be indicative of environmental conditions and change [4, 5]. Morphological traits relating to leaf shape and size have successfully been measured on dried specimens [6], but many traits related to leaf structure cannot be measured on dried samples. Leaf nutrient analyses (such as leaf carbon, nitrogen and phosphorus contents) require destructive analyses. Leaf nitrogen and phosphorus contents in particular can indicate increased nutrient supply in the soil [7], while also being correlated with competition-related traits such as leaf mass per area, leaf lifespan and leaf chlorophyll content [5]. Unfortunately, studying these important metrics for historic plant nutrition and fitness necessitates destructive sampling of irreplaceable herbarium specimens [8, 9]. The contrast between the importance of these leaf measurements and the currently available methods thus makes a non-destructive measurement method desirable.

One approach to extract data on leaf nutrients from herbarium specimens in a non-destructive way is the use of Near-Infrared Spectroscopy (NIRS). This method has proven itself in various ecological studies of e.g. herbivory defense traits in grasses [10], intraspecific leaf trait variability of forbs [11] and trees [12, 13]. NIRS emits near-infrared radiation onto a sample and then measures the full reflection spectrum. In a second step, this spectrum needs to be calibrated using the content of target chemicals in the sample. This reference calibration model can then be used to predict leaf nutrient contents from NIRS readings. Once a calibration dataset with data from destructive laboratory analytical methods as well as the spectral data model is created, predicting trait values of new samples only requires scanning with the NIRS device, which is a fast, cheap, and non-destructive process. Following the underlying theory, calibration models for chemical leaf compounds that are directly related to the overall leaf structure such as leaf carbon and nitrogen are most reliable [14], but leaf nutrients with low overall amounts in the leaf such as calcium and phosphorus can also be calibrated for [15]. Furthermore, traits like leaf dry matter content and specific leaf area can also be calibrated for due to their close relation with leaf nutrient contents [11]. In general, calibrations for target nutrients with overall lower contents in the leaf, like phosphorus, are less reliably calibrated for [16].

The fact that NIR spectroscopy is heavily influenced by surface characteristics of a given sample also poses a challenge: there is a possibility that readings are influenced by leaf shape and size, leaf surface characteristics like trichomes, cuticula layers, or, in the case of herbarium specimens, dust and insecticides. Scanning milled leaf samples can reduce the impact of these characteristics and improve calibration qualities [17]. In the case of herbarium specimens this would however again result in a destructive analysis. Other potential sources of error are treatments applied for long-term conservation of samples, which can include both freezing and drying [18]. Over one century, a typical herbarium specimen would have been repeatedly dried in an oven and frozen to prevent or actively suppress insect pests. Research on the effect of conservation treatments has found that long-term leaf powder storage does not affect nitrogen levels [19], but freezing and drying can both affect the leaf dry matter content as well as the leaf carbon content [20]. The latter study thus questions that the measurements on herbarium specimens accurately reflect the traits at the time of sampling.

In spite of the aforementioned challenges with using NIRS methods, there is an impressive potential for ecological studies, not least because of the vast size of herbarium collections and the potentially large statistical power of collection-based studies. Assuming robust calibration models and a comprehensive herbarium collection, one could carry out leaf trait analysis on samples covering a wide range of spatial and temporal scales. A recent study has demonstrated that physical and chemical leaf traits can be assessed with the NIRS analysis of pressed and dried leaves [21]. However, the challenge is now to not just provide a proof of concept of the general applicability of near-infrared reflectance spectroscopy for the analysis of herbarium specimens but also to understand possible confounding factors that are relevant when applying these methods to herbarium collections. The aim of our study is to investigate the impact of potentially confounding factors, such as the effects of long-term herbarium storage associated with pesticide or freezing applications on the spectra gathered from those samples. We have thus set the following objectives for our study:

1. Can plant responses to increased nitrogen and phosphorus input be captured equally well using regular wet lab analyses or NIRS?
2. Do long-term conservation methods typically employed in herbaria influence the prediction of leaf N content of leaf samples?
3. Can changes in leaf spectra be detected after three years of herbarium storage?

## Methods

To test the suitability of NIRS for the analysis of herbarium leaf traits, we carried out three experiments. In all experiments, an ASD FieldSpec 4 Wide-Res Spectroradiometer with a contact probe (Malvern Panalytical Ltd, Almelo, Netherlands) was used to gather near-infrared diffuse reflection spectral data. The FieldSpec probe was placed on the adaxial side and the widest part of the leaf, with the spectral regions measured reaching from 350 to 2500 nm, with an integration time of 8.5 ms. Care was taken to have as much as possible of the 20 mm diameter lens of the probe covered with leaf material. A white reference target (Zenith Lite Target, SphereOptics GmbH, Herrsching, Germany) was used to calibrate the device in regular intervals. All samples were scanned thrice on the same spot, and the three spectra were averaged for further analyses.

### Experiment 1: fertilization experiment

In the greenhouse at the Botanical Garden Halle, a fertilization experiment was carried out to determine (a) how well the addition of basic nitrogen fertilizer and phosphorus fertilizer could be measured in plant leaf tissue through both conventional (gas chromatography or digestion and spectroscopy, respectively) and NIRS-based measurements and (b) if the press-drying of leaf samples negatively impacted the accuracy of NIRS measurements.

Three species common to central European grasslands*, Centaurea jacea* L., *Plantago lanceolata* L. and *Poa annua* L. were grown from seeds provided by an agricultural supplier (Rieger-Hoffman GmbH, Blaufelden-Raboldshausen, Germany) in late 2020. These species were selected for their differing leaf shapes, with the intention of comparing the quality of spectral readings gained from broad, intermediate or thin leaves, respectively. After initial germination in a closed tray, 80 individuals of each species were planted in plastic pots filled with a 1-to-2 mixture of sand and sterilized loam in January of 2021. Additionally, around 30 spare seedlings from each species were set aside and grown as an untreated control group. A plastic saucer was placed under each individual pot to prevent spillover or mixing of fertilizer solutions. The pots were placed on tables in a climate-controlled cabin with constant environmental conditions: 12 h of full daylight being provided each day, an air temperature of 20 °C during the day and 10 °C during the night, and an air humidity of 50%. To prevent small-scale environmental gradients in the cabin from influencing the plant growth, pots were positioned on a grid on each bench, and then reshuffled randomly across all benches every two weeks. Following a two-week acclimation period, we started applying nitrogen fertilizer and phosphorus fertilizer in a factorial scheme. In units of kilogram per hectare per year, the levels were 5, 20, 100, 200 for nitrogen and 1, 4, 20, 40 for phosphorus, supplied as NH_3_NO_3_ and K_2_HPO_4_, respectively. The result is a 4 by 4 table where each phosphorus or nitrogen fertilization level is crossed with every other resulting in 16 different fertilization levels in total. In addition, the plants received additional micro- and macronutrients to ensure optimal growth conditions. For the exact composition of the fertilizer solutions, see Additional file 1: Tables S1 and S2. The nutrients were concentrated in a way that 15 ml of the respective fertilizer mixture was added to each pot once per week and the total annual target amount in kilogram per hectare would have been reached in one year. Additional tap water was applied to maintain soil moisture. The fertilization continued for four months, which was the point when the first plants started flowering. The pots thus received a fertilization that corresponded to one third of the annual amounts of the fertilization levels in kilogram per hectare per year mentioned above.

In May 2021, plants were cut off at ground level, and NIRS readings were taken for the largest leaf of each plant. The plants were then press-dried in the Herbarium Halle (HAL). After two weeks of drying, another set of NIRS readings were taken, and some plant matter was removed for use in further laboratory analysis. The plant matter was ground into fine powder in a grinder mill (MM 400, Retsch, Haan, DE) and used to determine the carbon and nitrogen contents of each sample chromatographically (Vario EL Cube, Elementar Analysensysteme, Langenselbold, DE). Another fraction of the powder was subjected to acid digestion with nitric acid, and the liquid samples were analysed for phosphorus content using an ion chromatograph (ICS-90 Dionex, Thermo Fischer Scientific, Waltham, USA). The data from the NIRS and laboratory were used in conjunction to create a calibration dataset as extensively described in the “Calibration models” section below.

### Experiment 2: effect of herbarium conservation

An additional experiment was carried out to test for the influence of the following leaf conservation methods that are typically employed in herbaria (Jörn Hentschel, Herbarium Haussknecht, private communication, June 2021) on NIRS analyses: freezing, drying, and pesticide spraying.

**Fig. 1:**
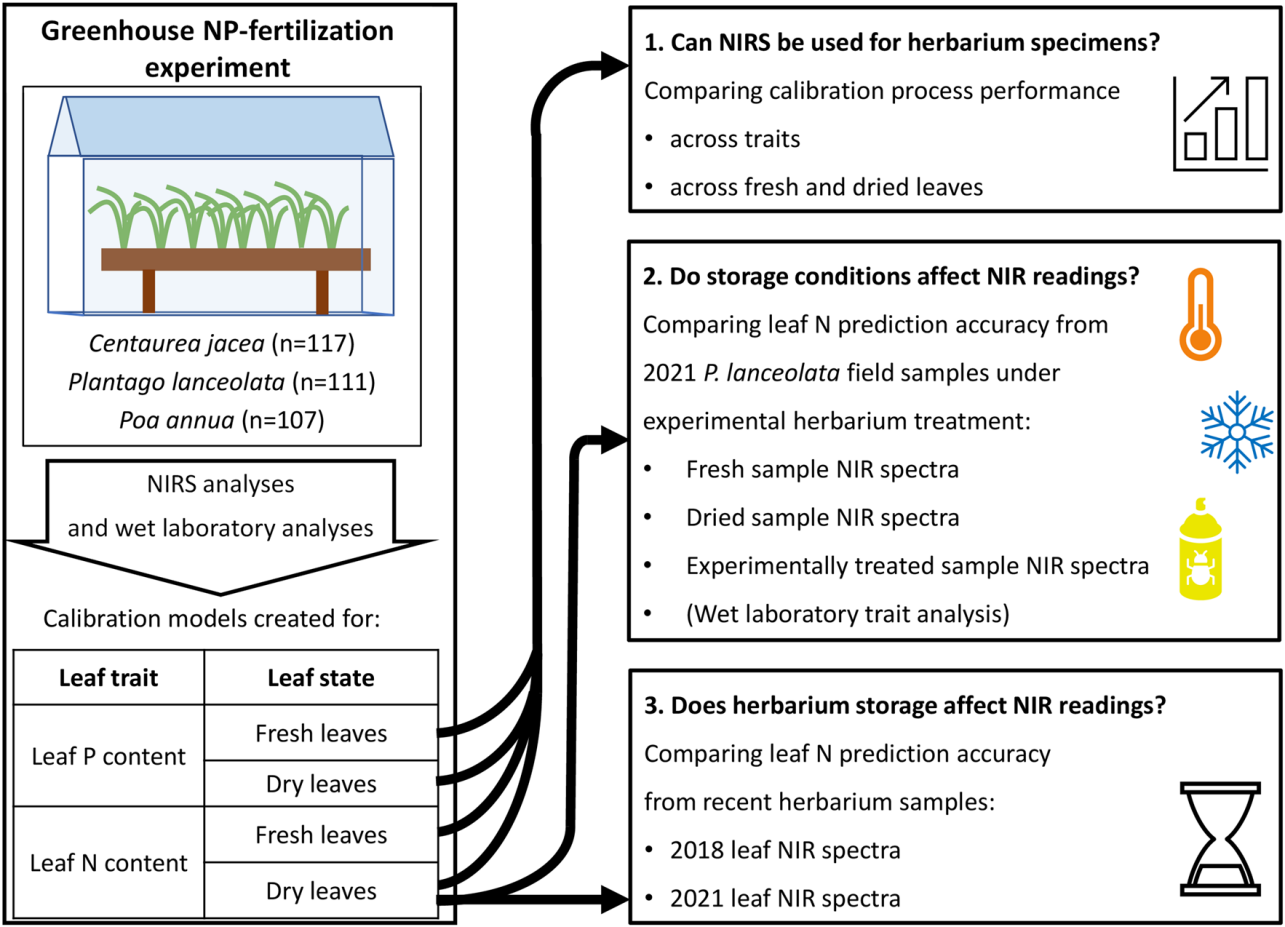
Graphical overview of the three different experiments. The fertilization experiment is used as baseline, with calibration models from that dataset used to answer the three research questions. Note that box number 1 on the right is still part of the fertilization experiment, but specifically refers to the analysis and comparison of performance metrics. Box number 2 represents the experiment simulating herbarium storage conditions, while box number 3 represents the experiment involving herbarium storage duration. Furthermore, all four calibration models created from greenhouse data are used to answer research question 1, while for the other two only the calibration model for leaf nitrogen content of dry leaves is used for analyses

For this, 40 fully grown, flowering specimens of *Plantago lanceolata* L. were collected from one population on a mesic grassland site in Jena, Germany, close to the Saale river (11.61009 E, 50.94427 N) in August of 2021. NIRS readings were taken of the fresh samples on the same day, after which the plants were press-dried in the Herbarium Haussknecht (JE). After drying, specimens were again scanned with the FieldSpec. Leaf tissue was then taken from the samples to measure the leaf carbon–nitrogen content using wet lab methods as described above for the laboratory analyses of Experiment 1. To simulate the effects of long-term storage and conservation treatments six different treatment groups combining freezing, drying and pesticide use were set up. The pesticide used consisted of a mix of permethrin and pyrethrine (Detmol-Flex, Frowein, Albstadt, Germany) which was liberally applied once to two of the treatment groups. The drying and freezing treatments each lasted 48 h, at + 60 °C and − 20 °C respectively, followed by a pause of at least 24 h to allow samples to return to room temperature. Table 1 provides an overview on the six treatment groups freezing, drying, pesticide, freezing and pesticide, freezing and drying and finally freezing, drying and pesticide use. NIRS readings were then again taken for each sample and predictions for leaf N content were calculated using the greenhouse calibration model for leaf nitrogen content of dried leaves (see Fig. 1), for the three different spectral datasets: fresh, dried and treated. In combination with regular C and N measurements from laboratory analyses, this allowed a direct comparison and measurement of the impact of the treatments on trait prediction accuracy.

**Table 1:** Overview table for the second experiment describing groups and respective treatments. Combinations were carried out by alternating freezing and drying treatments. Pesticide treatments were always carried out as last part of the sequence

Group	Treatment		
	Freezing	Drying	Pesticide
Freezing	X		
Drying		X	
Freezing + drying	X	X	
Freezing + pesticide	X		X
Freezing + drying + pesticide	X	X	X
Pesticide			X

### Experiment 3: herbarium storage duration

In this experiment we utilized samples from a Master thesis carried out in Halle (Saale), Germany, in 2018. The thesis focused on herbaceous and shrub species that were common both in the herbarium collection of the herbarium Halle (HAL) and in habitats of the Halle region. Specimens already present in the collection of the herbarium Halle were compared with specimens collected in the field in the years 2018 and 2019 using NIR spectroscopy. NIRS was used to measure leaf nitrogen and sulfur contents, and spectroscopy readings were taken off recent samples before adding them to the collection of that herbarium. Field samples were press-dried according to the local protocols. It is furthermore prescribed to freeze new samples for 2 days to prevent a contamination of the collection with insect pests. No other conservation treatments took place during that three-year period (Marcus Lehnert, Herbarium Halle, private communication, March 2023). We scanned these samples again in 2021 with the FieldSpec. Using the calibration model for nitrogen content of dried leaves created from the greenhouse dataset, predictions of leaf nitrogen content were carried out based on the readings taken from the same sample in 2018 and 2021 and compared (see Fig. 1).

### Calibration models

The datasets collected in these experiments were used to create separate calibration models for leaf nitrogen and leaf phosphorus contents. From the fertilization experiment, two calibration models for each trait were created, one derived from spectral readings on fresh leaves and another one on dried leaves.

Based on the ASD FieldSpec sensor specifications the spectra were spliced at preset locations according to the output of an ASD FieldSpec 4 Wide-Res. We used test-set validation to assess the calibration model quality. The spectra were split into a calibration and validation dataset using the Kennard-Stone algorithm [22], yielding 50% calibration and 50% validation spectra. We used an optimized partial least squares regression (PLSR) model included in the “plantspec” package [23] to create the calibration models. PLSR is a powerful analysis tool that excels in predicting chemical values from a set of many collinear and noisy variables [24], and it has proven useful as a suitable framework to create NIRS calibration models [25]. As the plantspec package does not involve the selection of combinations of different spectral ranges to optimize the calibration model, which is available in some commercial software, we implemented this procedure based on a framework by Proß et al. [26]. By selecting randomized regions of the spectra to be included in the model, with each iteration focusing on up to eight different selected spectral regions. From the repeated randomized models, the model exhibiting the best performance metrics (see “Statistical analysis” section below) was selected for use in further analysis. For an optimal model, the reference trait value should be normally distributed. In the case of the dataset derived from the fertilization experiment, the experimental design made a transformation of the reference nitrogen content with the natural logarithm necessary.

In order to improve the calibration model, we implemented a univariate outlier detection test based on F-statistics. Any samples presenting F-values exceeding the 99.9th percentile of the F-distribution were identified as possible outliers. These were subsequently removed after manual re-evaluation in instances where extreme outliers led to low model qualities.

To compare spectra derived from samples in experimental treatments, a calibration model was created for each set, consisting of a data table of laboratory data and a data table consisting of the hyperspectral data measured from the sample. Spectra derived from fresh and dried, or dried and experimentally treated leaves, were thus treated as separate spectral datasets. Accordingly, even if the laboratory reference data is the same, the two different “fresh” and “dried” spectral datasets will create two different calibration models. To compare and analyze the sample sets that underwent differing conservation treatments, leaf nitrogen content was predicted separately for each set of spectral data. To this end, the greenhouse leaf nitrogen calibration model for dried leaves was used (see Fig. 1), from here on referred to as the “reference calibration model”. For the R code used to carry out the calibrations and comparisons, see Additional file 2.

### Statistical analysis

To assess if plant responses to nitrogen and phosphorus fertilization can be captured equally well through wet lab methods, fresh and dried leaf NIR scans, we calculated R^2^ and root mean square error of prediction (RMSEP) as general performance metrics for the resulting calibration models, with ideal values of the former approaching 1 while ideal values for the latter approach 0. As such, here R^2^ and RMSEP values are used to compare values of a given trait as measured in the lab to those predicted by our calibration model. They are thus used as a shorthand for the quality of a calibration model, i.e., how accurately it manages to predict these trait values.

In the investigation of the influence of conservation methods used in herbaria on leaf trait predictions, we statistically compared the various treatment groups and conditions. Due to the heterogeneity of the datasets, different methods and tests were used to determine correlations and significant effects by group. Analyses of the results from experiment 2 were carried out using a Kruskal–Wallis test, with the predicted leaf nitrogen content as a dependent variable, and the state of the leaf the data was derived from (fresh leaf spectra, dried leaf spectra, treated leaf spectra, wet laboratory analyses) as independent variables. The same approach was used to test for the influence of different treatments on the predicted leaf nitrogen content: the predicted leaf nitrogen was used as dependent, the treatment group levels were used as independent variables. Where a Kruskal–Wallis test indicated significant differences between groups, Dunn’s test was used to determine which groups specifically differed.

To determine if a three-year herbarium storage duration caused changes in the measured NIR spectra, we used Pearson’s correlation test to quantify the similarities between the leaf nitrogen content from the same samples, as predicted based on either NIRS measurements carried out in 2018, 2021, or wet laboratory analyses. For the exact code employed to carry out these analyses, see Additional file 2.

The R programming language [27] was used for all analyses. Besides the already mentioned “plantspec” package [23], the packages “foreach” [28] and “doParallel” [29] were used to parallelize the calculations. The analysis and plotting were facilitated by the packages included in the “Tidyverse” [30].

## Results

**Fig. 2:**
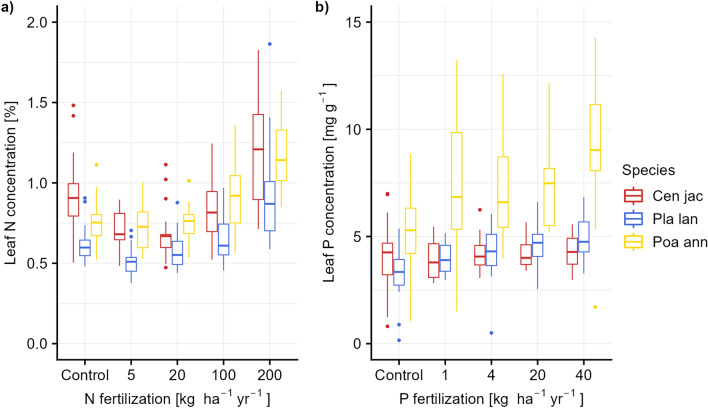
Leaf nutrient contents for nitrogen and phosphorus in relation to the studied plant species and the N- and P-fertilization in the fertilization experiment, based on wet-lab measurements. The color coding is red for *Centaurea jacea* L., blue for *Plantago lanceolata* L. and yellow for *Poa annua* L.

**Fig. 3:**
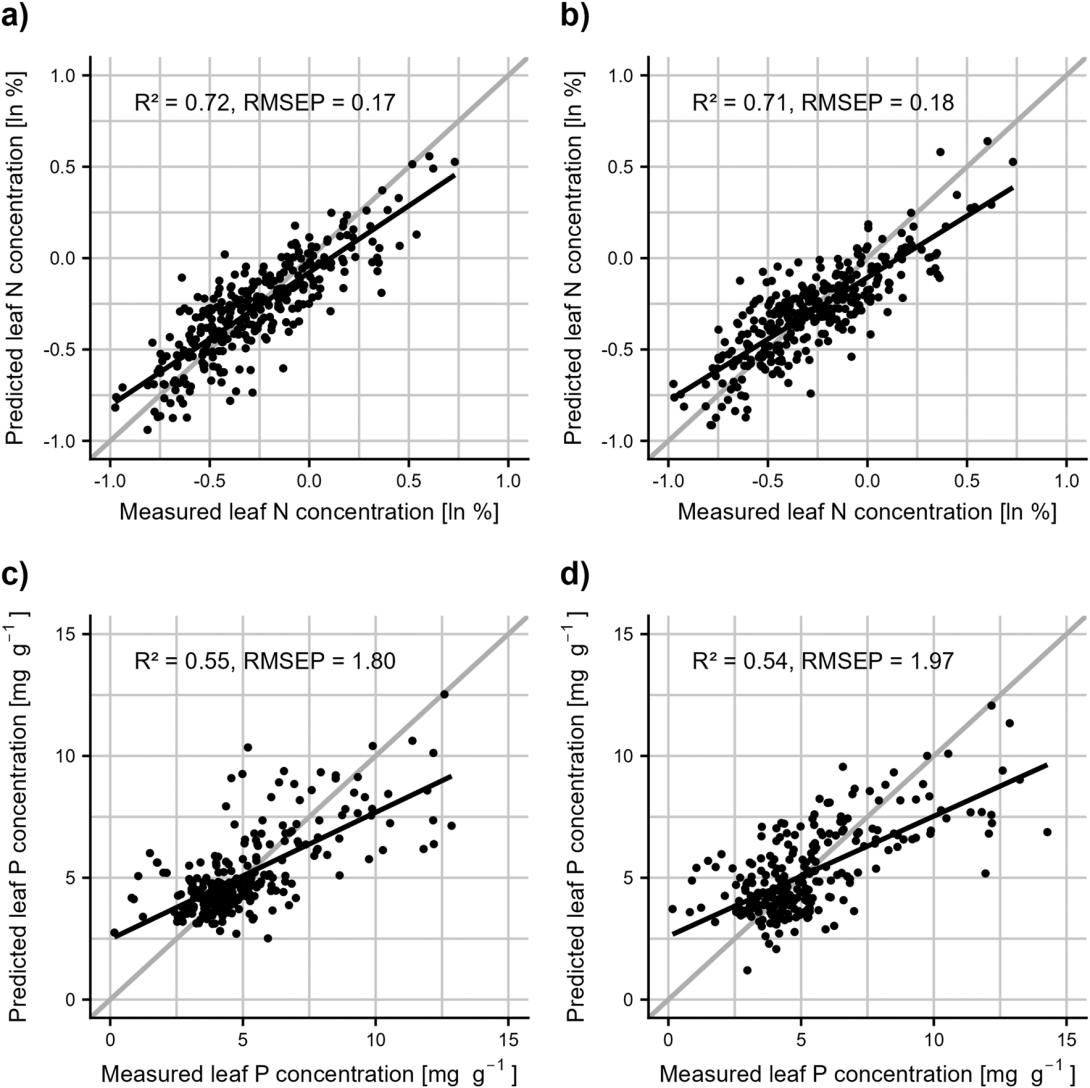
Model-predicted leaf nutrient contents versus lab-measured leaf nutrient contents for nitrogen and phosphorus as well as fresh and dried leaves based on the fertilization experiment data. Each plot represents one calibration model, each point represents one sample. R squared and root mean square error of prediction (RMSEP) are shown as measures of model quality. The black line is a simple linear model to visualize the deviation of the calibration from the idealized perfect fit represented by the grey line

The results of the data from the fertilization experiment show that effects of fertilization on leaf chemical composition were clearly detectable in the wet lab analyses (Fig. 2a, b): leaf nitrogen increased in a nonlinear fashion mirroring fertilization intensity, and so did leaf phosphorus (though less pronounced). Calibration models created for leaf nitrogen and phosphorus were compared across the two different leaf states of fresh and dried leaves. The performance metrics comparing measured to predicted trait values suggest that values of these two leaf states are highly correlated for both traits: the resulting models for the logarithm of leaf nitrogen content had an R^2^ of 0.72 and an Root Mean Squared Error of Prediction (RMSEP) of 0.17 for fresh leaves and an R^2^ of 0.71 and an RMSEP of 0.18 for dried leaves (Fig. 3a, b); the calibration model for leaf phosphorus content had an R^2^ of 0.55 and an RMSEP of 1.94 for fresh leaves and an R^2^ of 0.54 and an RMSEP of 9.4 for dry leaves (Fig. 3c, d). Prediction quality differed between species, with lower values for the grass *Poa annua* than for the two broad-leaved species in predicted leaf nitrogen content, and comparatively better, but still low values for leaf phosphorus content (see Additional file 1: Fig. S1, Table S3).

**Fig. 4:**
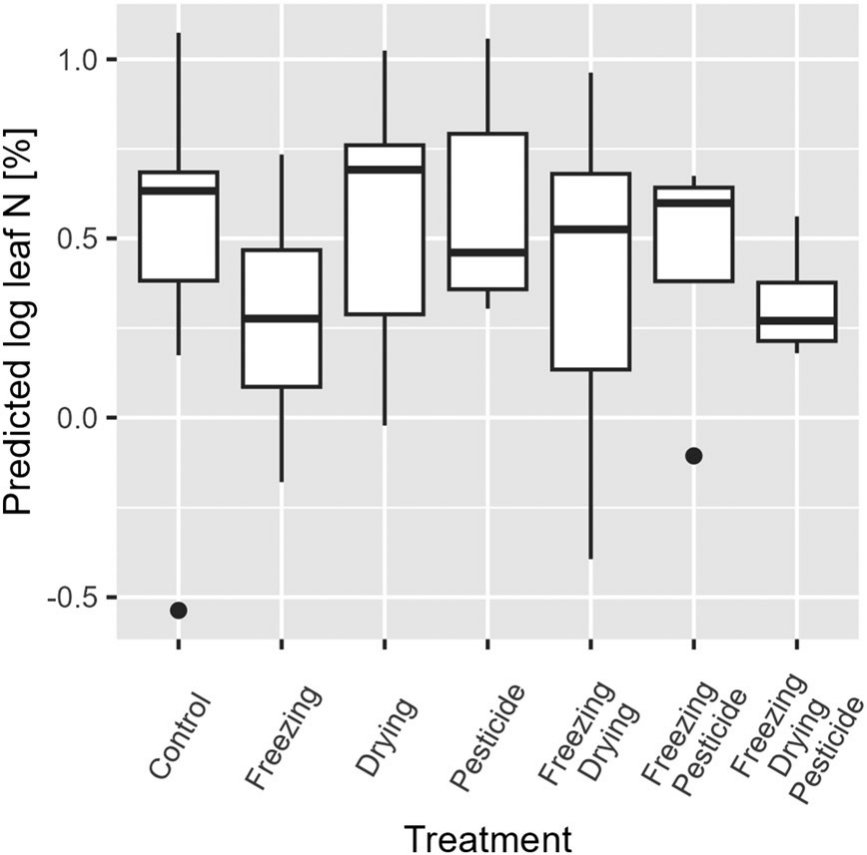
Predicted leaf nitrogen values of *Plantago lanceolata* L. samples in different treatment groups. Predictions are carried out by applying the leaf nitrogen-dried leaf calibration model from the fertilization experiment to NIR spectral data gathered of the samples after treatment

In the second experiment in which long-term herbarium storage was simulated through experimental treatments, there were no significant differences in the values predicted by the reference calibration model from the dried leaves before and after treatment. Predicted leaf nitrogen content did not significantly differ between treatment groups (Fig. 4, Χ^2^ = 3.30, df = 6, p = 0.771). Comparing the leaf trait values derived from both wet laboratory analyses and predictions based on spectra from fresh, dried, and treated leaves, a significant difference was found (X^2^ = 49.22, df = 3, p < 0.001), but this was driven by predictions based on fresh leaf spectra diverging strongly from the other two prediction groups and the laboratory reference values (Additional file 1: S1–S3). The quality of the calibration models created from the small dataset was low (R^2^ = 0.45, RMSEP = 0.28), for both the spectra from dried and treated leaves. The spectral lines of dried as well as dried and treated leaves also differed in their general shape and form. However, the experimental treatment of the leaves had no consistent effects on the spectra or the trait values predicted from them (Additional file 1: Fig. S2).

In the analysis of the effects of storage duration, two sets of comparisons were carried out. The first directly measured the correlation between the predicted leaf nitrogen values based on spectra gathered off the same sample set in 2018 and 2021. The correlation between the predicted leaf nitrogen values based on spectra from different years was low (Pearson’s Correlation Coefficient: 0.140). The second compared the predicted leaf nitrogen values based on 2018 and 2021 spectra with the actual laboratory measurements. The correlation here was also low, but the predicted trait values based on 2021 spectra proved themselves to be more highly correlated with the actual laboratory measurements (Pearson’s Correlation Coefficient: 0.326) than the predicted traits based on the 2018 spectra (Pearson’s Correlation Coefficient: 0.134).

Further data and statistical analyses from these two experiments can be found in Additional file 1: S4).

## Discussion

In the three experiments that were carried out, we corroborated the notion that NIRS has a high potential for the non-destructive analysis of leaf nutrient contents in herbarium specimens. We found no evidence for negative impacts of regular press-drying or more extensive conservation methods on the detectability of leaf functional traits through NIRS spectroscopy nor negative impact of storage duration.

In regard to our first research objective, we could confirm that high levels of fertilization have a detectable effect on measured leaf nutrients. In the fertilization experiment, the measured leaf nitrogen and phosphorus contents reflected those of the fertilization levels. This implies that plant samples including herbarium specimens allow tracking changes in soil nutrients and fertilization, which is a result in line with other studies tracking the direct impact of fertilization on leaf nutrient contents [7]. From dried greenhouse samples, calibration models could be derived for leaf nitrogen and phosphorus which were of comparable quality to another recent study investigating this method [21]. Only minimal differences in quality were observed compared to the models derived from fresh leaf spectra. While the calibration quality for leaf phosphorus was lower than for leaf nitrogen, this is also in line with previous studies and can be explained with the overall lower content of that element within the leaf [16]. The workflow is likely to yield even better results with more numerous and diverse data from the field, since PLSR models improve with larger sample sizes [24].

In the second experiment, we found no significant impact of storage conditions on the predicted leaf nitrogen. Furthermore, comparing predictions made from leaves in fresh, dried and treated states with the laboratory reference revealed that the fresh leaf spectra predictions constituted an outgroup, while the other three values were closely related. Our data therewith suggests that repeated conservation treatments do not bias leaf nutrient predictions by pushing the predicted values in one particular direction. Our results thus mirror the conclusion of [19] who found no effect of drying and long storage on the chemical composition of plant samples. We expect this result to also hold true for phosphorus, because P and N contents are similarly dependent on the overall molecular composition, which also includes the organic components of the leaf. As long as neither P, nor N is released from the leaf at temperatures above 60 °C and the leaf is not partly decomposed because of moist storage conditions, the context of the NIRS measurements would also not change, and thus, measurements on old plant material would be reliable. We were not able to discern a decrease in leaf nutrient contents as described by Portillo and Estrada [20], possibly because that study found the most significant effects while drying isoprenoid-rich conifer species at higher temperatures. Caution might thus be needed when including such species in future NIRS-based herbarium studies. The spectral lines derived from the treated samples were scattered randomly but without a clear pattern regarding the treatment group. This could indicate that the sample size (five individual samples per group) was too small to discern a pattern, and that small intraspecific variations in leaf shape, surface and health overshadowed the effects of the experimental treatment.

The data relating to our third research question regarding the herbarium storage duration showed some mixed results. Leaf nitrogen values predicted using the reference calibration model differed strongly from the actual laboratory data. However, the difference in species composition between the reference calibration model and the actual spectra was large. As such it is possible that the spectra from the previous project’s samples are outside the calibration range. Additionally, there was only a small correlation between predicted values based on 2018 and 2021 spectral data, with the predictions based on the 2021 however showing a higher correlation with the laboratory data. The slight increase in correlation of the 2021 spectra could be due to 2018 readings having been carried out by a different operator, with resulting changes in the way spectra are gathered, e.g. through the placement of the sensor head on the leaf, frequency of white calibration routines, etc.

## Conclusions

Taken together, our experiments indicate that NIRS is a valid and useful method to determine changes in leaf nitrogen and phosphorus contents of herbarium specimens induced by environmental changes. A good calibration model is necessary which includes enough species and covers enough trait variance to cover the expected variability. Furthermore, in cooperative projects care should be taken to use a consistent scanning protocol across all locations and operators to prevent noise being introduced into the underlying spectral dataset. Herbarium storage and conservation treatments do not appear to significantly influence the spectral data by themselves. A wider application of this approach to historical functional trait studies is thus possible. A combination of a well-catalogued herbarium, a portable NIR spectroscope and a robust calibration model are thus a powerful toolset to investigate past and ongoing changes in functional traits in a fast and economically parsimonious manner.

### Supplementary Information

**Additional file 1: **Additional experimental and statistical information for the manuscript “Using Near-Infrared Spectroscopy to predict nitrogen and phosphorus contents of herbarium specimens under different storage conditions”. This file contains information which can be used to augment the understanding of the main manuscript methods and results sections.

**Additional file 2: **R code and data for the manuscript “Using Near-Infrared Spectroscopy to predict nitrogen and phosphorus contents of herbarium specimens under different storage conditions”. This file contains two parts. First, the R-code_supporting_information.R, which can be used to recreate statistics and plots presented in this publication. Second, the “Basic_code”, which can be used for the creation and analysis of NIRS calibrations, as well as two data tables as formatting examples.

## Data Availability

The datasets supporting the conclusions of this article are included within the article and its additional files. Additional data tables and the server calibration code are available on request from the corresponding author, Paul Kühn, at paul.kuehn@uni-jena.de.
