# Supplementary material for: Using near-infrared spectroscopy to predict nitrogen and phosphorus concentrations of herbarium specimens under different storage conditions
Source: Plant Methods. 2024 Feb 1;20:19. doi: 10.1186/s13007-024-01146-x (PMC10835964; doi:10.1186/s13007-024-01146-x)
Supplement: Supplementary file 1 — Additional file 1: Additional experimental and statistical information for the manuscript “Using Near-Infrared Spectroscopy to predict nitrogen and phosphorus contents of herbarium specimens under different storage conditions”. This file contains information which can be used to augment the understanding of the main manuscript methods and results sections. [file 13007_2024_1146_MOESM1_ESM.docx]

**Additional file 1**

*Methods*

| Base solution type | Preparation |
| --- | --- |
| Nitrogen base solution | 16.352 g NH_4_NO_3_ in 1000 ml tap water |
| Phosphorus base solution | 6.433 g K_2_HPO_4_ in 1000 ml tap water |
| Calcium base solution | 29.283 g CaCl_2_ · 2 H_2_0 in 1000 ml tap water |
| Potassium phosphorus compensation | 2.683 g KCl in 1000 ml tap water |
| Potassium base solution | 1.707 g KCl in 1000 ml tap water |
| Magnesium base solution | - 1. MgSO_4_ · 7 H_2_0 in 1000 ml tap water |

Table S1: Reference table for the fertilizer base solutions mixtures of the greenhouse experiment.

| N-Level | P-Level | N | P | Ca | K for P-Compensation | K additional | K total | Mg |  | Total |
| --- | --- | --- | --- | --- | --- | --- | --- | --- | --- | --- |
| kg/ha per Year | kg/ha per Year | ml | ml | ml | ml | ml | ml | ml |  | ml |
|  |  |  |  |  |  |  |  |  |  |  |
| 5 | 1 | 1 | 1 | 40 | 40 | 40 | 80 | 40 |  | 162 |
| 20 | 1 | 4 | 1 | 37 | 40 | 40 | 80 | 40 |  | 162 |
| 100 | 1 | 20 | 1 | 21 | 40 | 40 | 80 | 40 |  | 162 |
| 200 | 1 | 40 | 1 | 0 | 40 | 40 | 80 | 40 |  | 161 |
| 5 | 4 | 1 | 4 | 40 | 37 | 40 | 77 | 40 |  | 162 |
| 20 | 4 | 4 | 4 | 37 | 37 | 40 | 77 | 40 |  | 162 |
| 100 | 4 | 20 | 4 | 21 | 37 | 40 | 77 | 40 |  | 161 |
| 200 | 4 | 40 | 4 | 0 | 37 | 40 | 77 | 40 |  | 161 |
| 5 | 20 | 1 | 20 | 40 | 21 | 40 | 61 | 40 |  | 162 |
| 20 | 20 | 4 | 20 | 37 | 21 | 40 | 61 | 40 |  | 161 |
| 100 | 20 | 20 | 20 | 21 | 21 | 40 | 61 | 40 |  | 161 |
| 200 | 20 | 40 | 20 | 0 | 21 | 40 | 61 | 40 |  | 161 |
| 5 | 40 | 1 | 40 | 40 | 0 | 40 | 40 | 40 |  | 161 |
| 20 | 40 | 4 | 40 | 37 | 0 | 40 | 40 | 40 |  | 161 |
| 100 | 40 | 20 | 40 | 21 | 0 | 40 | 40 | 40 |  | 161 |
| 200 | 40 | 40 | 40 | 0 | 0 | 40 | 40 | 40 |  | 160 |

Table S2: Reference table of the volume of fertilizer base solutions applied to the different groups of the greenhouse experiment.

*Results*

Table S3: Correlation table for log leaf nitrogen content based on dried leaf spectra and laboratory reference value per species, as carried out in R.

| Species | Correlation of model-predicted log leaf nitrogen value and reference value |
| --- | --- |
| Centaurea jacea | 0.857 |
| Plantago lanceolata | 0.781 |
| Poa annua | 0.573 |


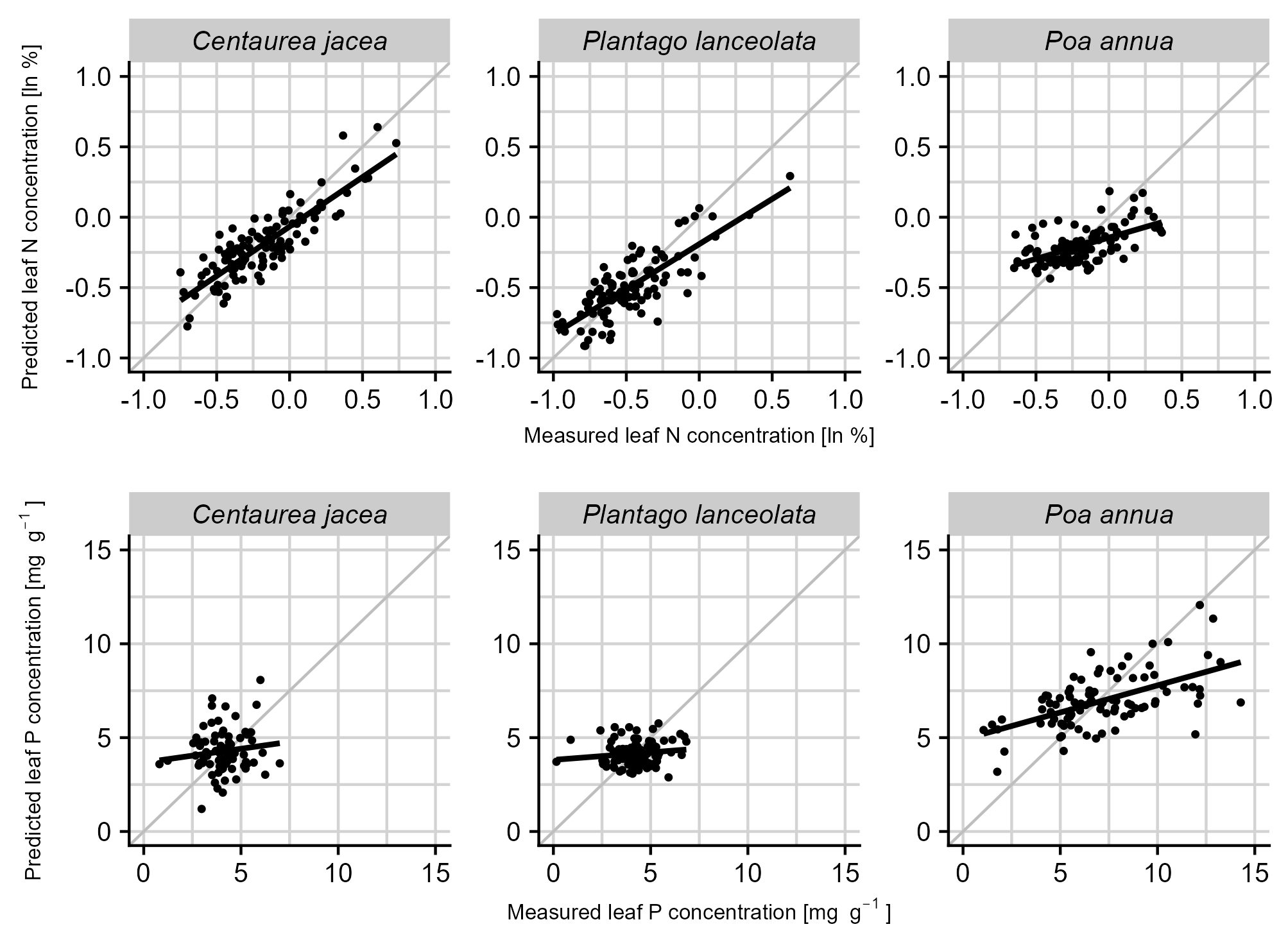


Figure S1: Model-predicted leaf nutrient concentrations versus lab-measured leaf nutrient concentrations for nitrogen and phosphorus as well as fresh and dried leaves based on the fertilization experiment data, shown separately for each species. Each plot represents the subset of samples from that species within the calibration model, each point represents one sample. The black line is a simple linear model to visualize the deviation of the calibration from the idealized perfect fit represented by the grey line.


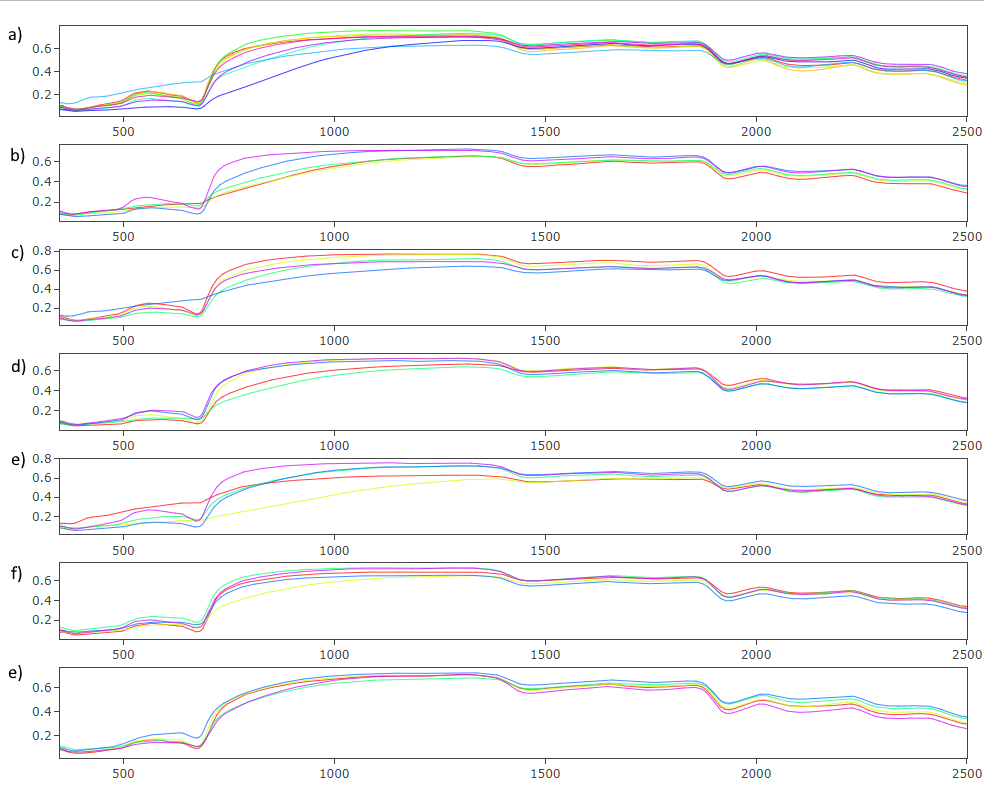


Figure S2: Reflectance spectra from the storage condition experiment, with the wavelength indicated in nm on the x-axis. The seven plots are for the different experimental treatments of a) control, b) freezing, c) drying, d) pesticide application, e) freezing and drying, f) freezing and pesticide application and e) freezing, drying and pesticide application. The different colors indicate different replicates.

S1: Statistical analysis of treated samples for differences between experimental treatment groups. Due to non-normal distribution of sample traits, a Kruskal-Wallis test is used.

Kruskal-Wallis rank sum test

data: predicted log leaf nitrogen by experimental treatment group

Kruskal-Wallis chi-squared = 3.2968, df = 6, p-value = 0.7708

S2: Statistical analysis of treated samples for leaf nitrogen content of leaves as either predicted from fresh (F), dried (D) and treated (T) leaves or measured in the lab (L). Due to non-normal distribution of sample traits, a Kruskal-Wallis test is used.

Kruskal-Wallis rank sum test

data: logleafN by status

Kruskal-Wallis chi-squared = 49.216, df = 3, p-value = 1.173e-10

S3: To determine the grouping underlying the significant differences between leaf states, Dunn’s test was carried out

Dunn (1964) Kruskal-Wallis multiple comparison

p-values adjusted with the Bonferroni method.

Comparison Z P.unadj P.adj

1 D - F 5.47460483 0.0000000438488996 0.000000263093398

2 D - L -0.03044437 0.9757126575385613 1.000000000000000

3 F - L -5.50462339 0.0000000369958276 0.000000221974965

4 D - T -0.75755797 0.4487156468525377 1.000000000000000

5 F - T -6.18511736 0.0000000006205629 0.000000003723377

1. L - T -0.72732284 0.4670282067755602 1.000000000000000

S4: Statistical models and results for the experiment on a three-year herbarium storage period as carried out in R.

Pearson's product-moment correlation

Data: Predicted log leaf nitrogen based on 2021 spectra and lab measurements

t = 2.8506, df = 70, p-value = 0.00573

alternative hypothesis: true correlation is not equal to 0

95 percent confidence interval:

0.09816852 0.51564533

sample estimates:

cor

0.3225025

Data: Predicted log leaf nitrogen based on 2018 spectra and lab measurements

t = 1.1424, df = 70, p-value = 0.2572

alternative hypothesis: true correlation is not equal to 0

95 percent confidence interval:

-0.09950284 0.35580159

sample estimates:

cor

0.1352842

lm (Lab measured log leaf N ~ predicted log leaf N based on 2021 spectra +

predicted log leaf N based on 2018 spectra)

Residuals:

Min 1Q Median 3Q Max

-0.64320 -0.22867 -0.01404 0.17258 0.84203

Coefficients:

Estimate Std. Error t value Pr(>|t|)

(Intercept) 0.65696 0.10350 6.347 1.98e-08 ***

Predmat_wide$logleafN_prediction_2021 0.13196 0.04884 2.702 0.00867 **

Predmat_wide$logleafN_prediction_2018 0.05175 0.06476 0.799 0.42697

---

Signif. codes: 0 ‘***’ 0.001 ‘**’ 0.01 ‘*’ 0.05 ‘.’ 0.1 ‘ ’ 1

Residual standard error: 0.3111 on 69 degrees of freedom

Multiple R-squared: 0.1122, Adjusted R-squared: 0.08649

F-statistic: 4.361 on 2 and 69 DF, p-value: 0.01646
